# Detection of endogenous circadian rhythms of clock gene mRNA expression in mouse lung tissue using slice cultures

**DOI:** 10.1016/j.xpro.2023.102280

**Published:** 2023-05-06

**Authors:** Ritsuko Matsumura, Koichi Node, Makoto Akashi

**Affiliations:** 1 The Research Institute for Time Studies, Yamaguchi University, 1677-1 Yoshida, Yamaguchi, Yamaguchi 753-8511, Japan; 2 Department of Cardiovascular Medicine, Saga University, 5-1-1 Nabeshima, Saga, Saga 849-8501, Japan

**Keywords:** Cell culture, Cell isolation, Gene Expression, Model Organisms, Tissue Engineering

## Abstract

Detection of endogenous circadian rhythms in clock gene mRNA expression requires that mice be sacrificed at regular time intervals over one or more days. This protocol uses culture tissue slices obtained from a single mouse to collect time-course samples. We describe the procedure from preparation of lung slices to rhythmicity analysis of mRNA expression, including details to create handmade culture inserts. This protocol is useful for many mammalian biological clock researchers because it allows a decrease in animal sacrifice.

For complete details on the use and execution of this protocol, please refer to Matsumura et al. (2022).^1^

## Highlights

• Harvesting time-course samples prepared by tissue slice culture
• RNA extraction and cDNA synthesis for real-time quantitative PCR
• Rhythmicity analysis of mRNA expression of clock genes

## Abstract (editor highlights)

Publisher’s note: Undertaking any experimental protocol requires adherence to local institutional guidelines for laboratory safety and ethics.

## Before you begin

In this protocol, tissue slices in culture are harvested at regular time intervals over a period of one or more days, RNA is extracted, and the expression levels of clock genes are measured to detect the circadian rhythm of the peripheral clock. This protocol repurposes and modifies the tissue slice culture method used for real-time measurement of the circadian bioluminescence rhythm of the luciferase-fused clock gene (e.g., *Per2*^*Luc,*2^) harbored by mice.^1^^,^^2^ The procedure described below is performed at a 6-hourly time point over two days. The duration and time point intervals can be changed, however, and should be set according to the purpose of your study. Hughes et al.^3^ will be helpful with your experiment design. However, note the number of tissue slices that can be prepared from one mouse and the time required to obtain them. To ensure a sufficient amount of RNA, about ten slices per time point are required. If slice collection takes too long, the viability of the slices decreases. In our experience, 4- to 8-h intervals for one to two days is practical. This protocol may be applied to any strain of mice. Here, mice with the C57BL/6 genetic background^4^ are used. In our experiments, preparing a sufficient number of slices from the lung of one mouse for a duplicate of ten time points in total (6-h intervals over a two-day time course) was successful.

### Institutional permissions

All animal procedures described in this protocol have been approved by the Institutional Animal Use Committee of Yamaguchi University. Obtain approval from the regulatory authority of your institution before beginning experiments.

### Preparation of adult mice

**Timing: > 2 weeks**

1. Estimate the number of mice needed according to your experimental plan.
2. Prepare mice of a consistent age in weeks for the purpose of the experiment by breeding or purchasing from a vendor.
  a. House mice in an animal facility with a 12-h light/dark cycle until experiments begin. Maintain the room temperature at 23°C, and provide food and water ad libitum.
    ***Alternatives:*** Acclimate mice obtained from a vendor to these conditions for at least one week.
  b. Mice of the appropriate age are ready for the experiment.
    ***Note:*** Although mice can be used at any age, if obtained by breeding, the process takes at least three months.

### Creating handmade tissue culture inserts

**Timing: 1 day**

A culture insert is used to culture the slices. Commercially available inserts (Merck Millipore, Cat# PICM0RG50) can be used, but handmade inserts are also suitable, and allow for easier manipulation during slice collection. The protocol below describes the use of a handmade insert.

**Figure 1:**
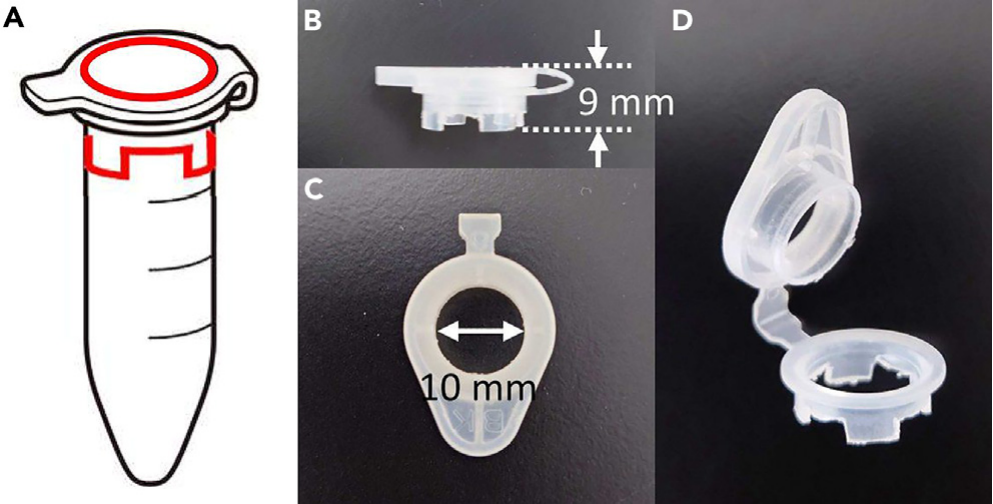
Overview of the handmade culture insert frame (A) Location of cut lines (solid red line) on the complete 5 mL tube. (B) Photo of the side of the frame. The height is 9 mm. (C) Photo from the top. The diameter of the hole is 10 mm. (D) Overall view of the finished frame. The frame snaps the membrane between the lid with the hole and the circular frame with the stands.

***Note:*** Make sure that it is horizontal when standing. Frames can be reused repeatedly by washing and autoclaving after use.

**Figure 2:**
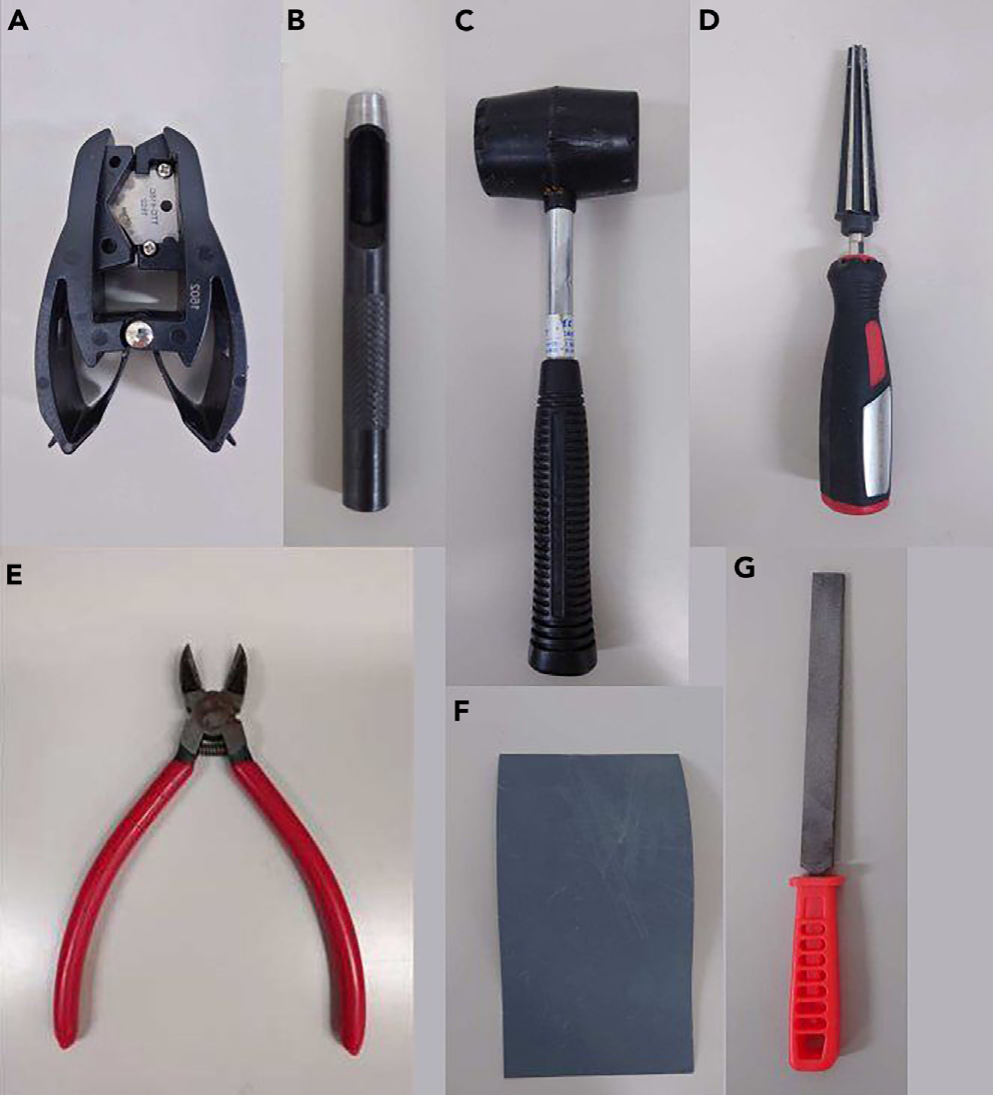
Seven tools used to create the handmade culture insert frames (A) Tube cutter, used to cut the tube into rounds. (B and C) (B) 8-mm diameter punch, used with a hammer (C) to make the hole. (D) Tapered reamer, used to widen the size of the hole. (E) Cutting nippers, used to form the holes and legs of the frame. (F and G) Sandpaper (Grit size, 1500) (F) and a file (G), used to adjust the height of the frame and to smooth any cut surface. See the key resources table for more information about A, B and D. The scale of the photos varies.
  b. Cut a 5-mL test tube with a pipe cutter at 9 mm from the top edge. First cut to half of the circle, then invert the tube and cut the other half (Figure 3A).

3. Cut out frames of the insert from 5 mL test tubes (Figure 1).
  a. Prepare tools (Figure 2).

**Figure 3:**
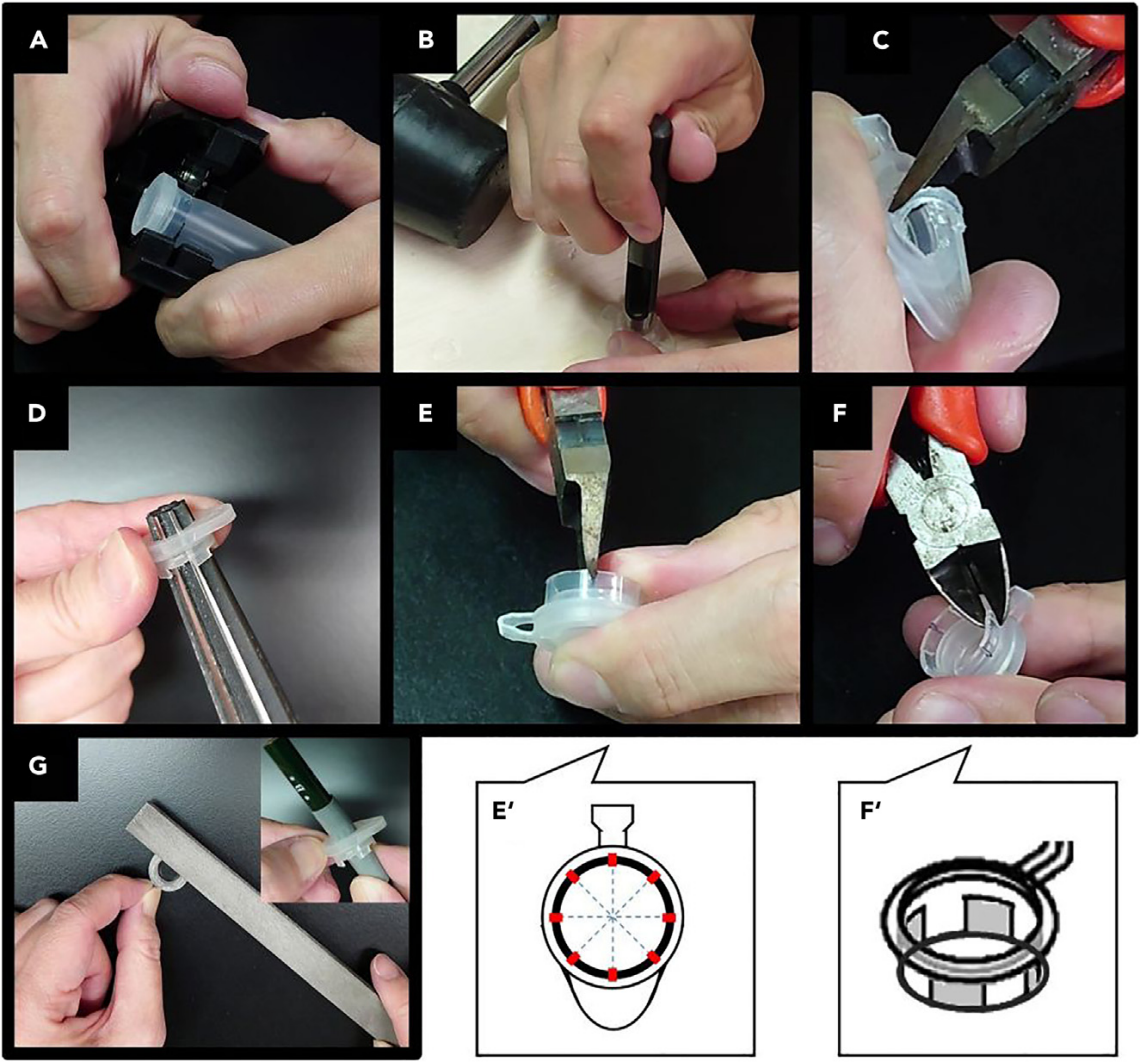
Making frames for culture inserts from 5 mL test tubes (A) Cut a 5-mL test tube with a pipe cutter at 9 mm from the top edge. (B) Make a hole in the lid with an 8-mm diameter punch and a hammer. (C) Widen the hole with cutting nippers. (D) Widen the hole to 10 mm in diameter while shaping the cross-section of the hole with a tapered reamer. (E and F) Shape out the stand part. Make 8 even cuts with the nippers (E′, red solid line) and remove the unnecessary parts (F′, gray parts) with the nippers. (G) Shave the stands with a file so that the frame becomes horizontal. Further, smooth the cut surface with sandpaper. A pencil covered with sandpaper is used to smooth the cut surface of the hole (inner picture in (G)).
  c. Make a hole in the lid with an 8-mm diameter punch and a hammer (Figure 3B).
  d. Widen the hole roughly with nippers until the size the tapered reamer fit through the hole (Figure 3C).
  e. Widen the hole to 10 mm in diameter while shaping the cross-section of the hole with a tapered reamer (Figure 3D).
  f. Shape out the stand part. Make 8 even cuts with nippers (Figure 8E', red solid line) and remove the unnecessary parts (Figure 3F', gray parts) with nippers (Figures 3E and 3F).
  g. Shave the stands with a file so that the frame gets horizontal. Further, smooth the cut surface with sandpaper (Figure 3G).

**Figure 4:**
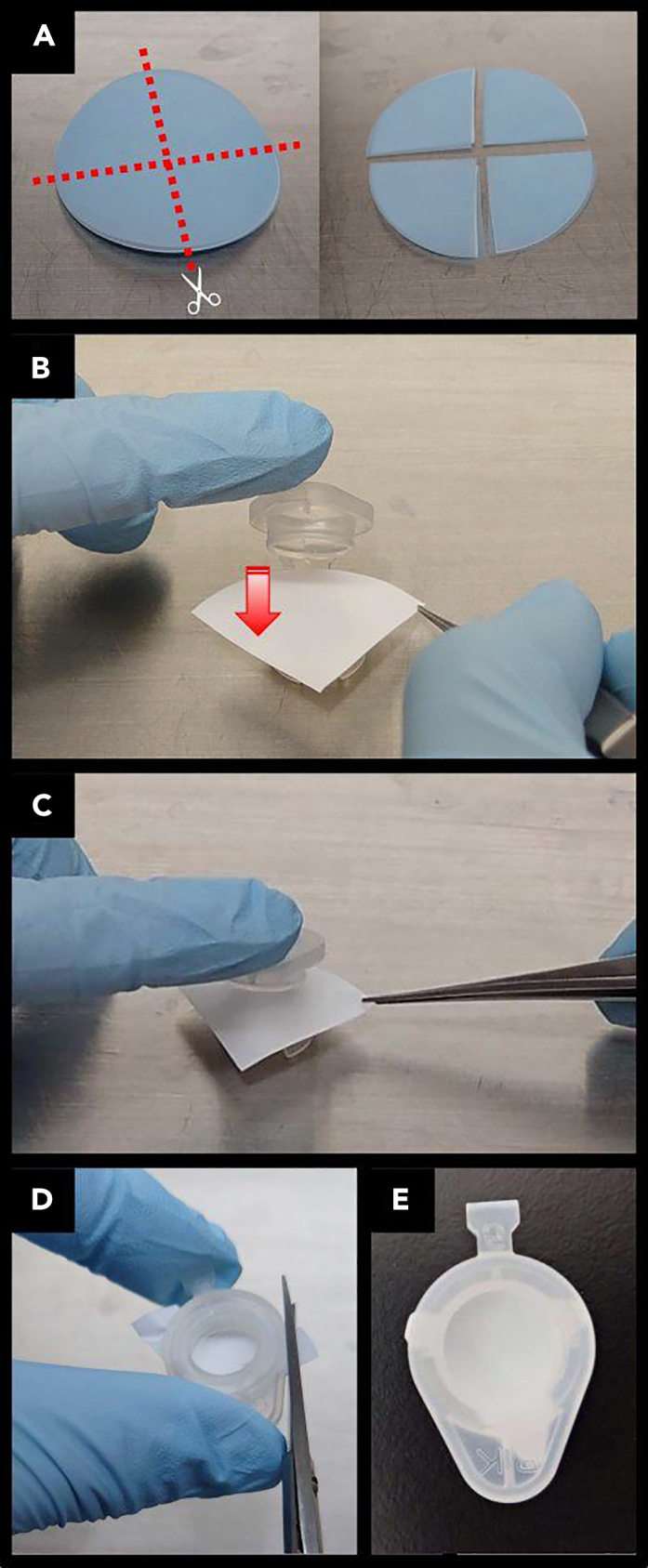
Setting the membrane on the handmade culture insert frame (A) Divide a sheet of membrane into four equal parts. As supplied, the membrane is sandwiched between blue protecting sheets. (B and C) Set one of the membrane parts on the frame by snapping the lid over it. (D) Cut off any membrane protruding from around the exterior of the frame. (E) The handmade culture insert is ready to use.

4. Autoclave the frames.
5. Set membrane (Merck Millipore, Cat#JHWP04700) on the frames (Figure 4).
  a. Divide a sheet of membrane into four equal parts in a sterile environment (Figure 4A) and set one of the parts on the frame by snapping the lid over it (Figures 4B and 4C).
  b. Cut off any membrane protruding out from around the exterior of the frame (Figure 4D).

## Key resources table

**Table:** 

REAGENT or RESOURCE	SOURCE	IDENTIFIER
**Chemicals, peptides, and recombinant proteins**		

DMEM	Sigma-Aldrich	Cat# D2902
Sodium bicarbonate solution	Sigma-Aldrich	Cat# S8761
HEPES buffer solution	Nacalai	Cat# 17557-94
D-(+)-Glucose solution	Sigma-Aldrich	Cat# G8769
Penicillin-streptomycin solution (×50)	FUJIFILM Wako Chemicals	Cat# 164-25251
Fetal bovine serum	Sigma-Aldrich	Cat# 173012
Dexamethasone (DEX)	Sigma-Aldrich	Cat# D4902
Power SYBR Green Master Mix	Thermo Fisher Scientific	Cat# 4367659
RLT buffer	Qiagen	Cat# 79216

**Critical commercial assays**		

RNeasy Mini RNA isolation kit (contains RLT buffer)	Qiagen	Cat# 74106
QIAshredder	Qiagen	Cat# 79656
High Capacity cDNA Reverse Transcription Kit	Thermo Fisher Scientific	Cat# 4368814

**Experimental models: Organisms/strains**		

*PER2*^*Luc*^	Yoo et al.^4^	N/A
*Cry1 E-box1*^m1/m1^*Cry2 E-box*2^Δ/Δ^	Matsumura et al.^1^	N/A

**Oligonucleotides**		

Real time PCR primers used in this study, see Table S1	This paper	N/A

**Software and algorithms**		

Cosinor	Refinetti	https://www.circadian.org/softwar.html

**Other**		

5-mL test tube	INA·OPTIKA	Cat# ST-500
Tube Cutter (4 - 16 mm)	TRUSCO	Cat# TTC-416
Tapered Reamer (10–22 mm)	Bigman	Cat# BTR-2
Hole Punch (8 mm)	Takagi	N/A
Non-treated culture dishes (φ 35 mm)	IWAKI	Cat# 1000-35
Tissue Chopper	McIlwain	Cat# 51350
BioMasher II (disposable homogenizer)	Nippi	Cat# 320102
Millicell	Merck Millipore	Cat# PICM0RG50
Omnipore Membrane Filter	Merck Millipore	Cat# JHWP04700
Real Time PCR System	Applied Biosystems	PRISM7300

## Materials and equipment

• Reagents for slice culture and circadian rhythm synchronization.

**Table:** Basal tissue slice culture media [FBS (-)]

Reagent	Final concentration	Amount
DMEM (powder)	1%	10 g
Sodium bicarbonate solution (7.5%)	0.0353%	4.7 mL
HEPES buffer solution (1 M)	10 mM	10.0 mL
D-(+)-glucose solution (45%)	0.351% (19.5 mM)	7.8 mL
Penicillin-streptomycin solution (×50)	×0.5	10.0 mL
ddH2O	N/A	Up to 1000 mL
**Total**		**1 L**

Sterilize the media using a 0.22-μm filter; store at 4°C for up to one month.

***Alternatives:*** In principle, all reagents listed in the table above can be substituted with equivalent items from other suppliers.

• 100 μM Dexamethasone: Dissolve dexamethasone (Sigma-Aldrich, Cat# D4902) in DMSO to prepare a 100 mM stock solution. Dilute it to 100 μM in saline. Store at −20°C for up to six months.
• Weighing paper used for cutting out slices: Wrap the weighing paper, cut to fit the shape of the circular stainless-steel table of the chopper with aluminum foil, and sterilize it.
• Equipment setup and recipe for real-time quantitative PCR (RT-qPCR).

**Table:** Equipment setup for RT-q PCR

Steps	Temperature	Time	Cycles
Initial Denaturation	50°C	2 min	1
	95°C	10 min	1
Denaturation	95°C	15 s	40
Annealing	60°C	1 min	
Extension			
Dissociation	95°C	15 s	1
	60°C	30 s	1
	95°C	15 s	1

**Table:** Recipe for RT-qPCR

Reagent	Final concentration	Amount
Forward primer (3 μM)	0.3 μM	2.5 μL
Reverse primer (3 μM)	0.3 μM	2.5 μL
SYBR master mix	N/A	12.5 μL
Nuclease-free water	N/A	2.5 μL
Diluted cDNA	N/A	5 μL
**Total**	N/A	25 μL

***Alternatives:*** SYBR master mix is available from many vendors. Select a suitable mix for your real-time PCR detection system. TaqMan is an option, but if used, the TaqMan probe must be prepared in addition to the primer set. More information about the TaqMan probe can be provided by the TaqMan reagent vendor.

## Step-by-step method details

The protocol below shows the procedure for a time course of 2 days (10 time points) at 6-h intervals.

### Preparation of culture media

**Timing: 1–2 h**

This section describes how to prepare the medium and dispense it into dishes. To streamline the process, prepare media for washing and re-culture for use after synchronizing stimulation with DEX as well as media for pre-culture at the same time. You can do this on the day of lung sectioning (Day 1 below) or the day before. If prepared the day before, store the medium at 4°C in a sealed container to prevent evaporation before use.

1. Add FBS (Sigma SIGMA, Cat#173012) to the basal tissue slice culture media at a ratio of 1:10.

***Note:*** There is no problem using FBS used for cell culture.

2. Dispense the FBS-supplemented culture media at 1.8 mL per non-treated 35 mm dish.

***Note:*** The number of dishes is twice that of the total needed in the experiment because they will be used for both pre-culture and re-culture.

3. Dispense the basal culture media at 1.8 mL per 35 mm dish for washing.
4. Put the inserts in the dishes with media for pre-culture (no need for washing and re-culture).

***Alternatives:*** If you use commercially available culture inserts (Merck Millipore, Cat# PICM0RG50) dispense 1.2 mL media per dish.

***Note:*** One slice on a culture insert of liver, pancreas, aorta, and white adipose tissue other than lung can also be cultured in this culture media.^1^^,^^2^ Therefore, this experimental protocol can be applied to tissues other than lung. However, note that we tested this protocol using liver, and the detection of circadian rhythm was more stable in lung than in liver.

### Day 1: Lung sectioning and slicing

**Timing: < 1 h**

**Figure 5:**
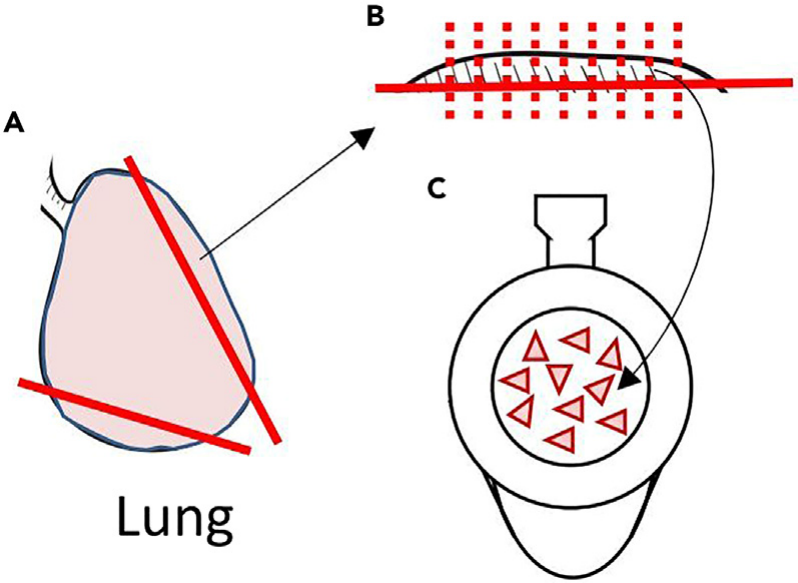
Schematic diagram of the lung slice preparation process (A–C) Cut out a portion of the lung as shown by the red line in (A) and make slices with a chopper (red dotted line shown in B). Place about 10 slices on the membrane of one insert (C).

On Day 1, lung slices are prepared and culture begins (Figure 5). Conduct this procedure as quickly as possible to prevent deterioration of the slices.

**Figure 6:**
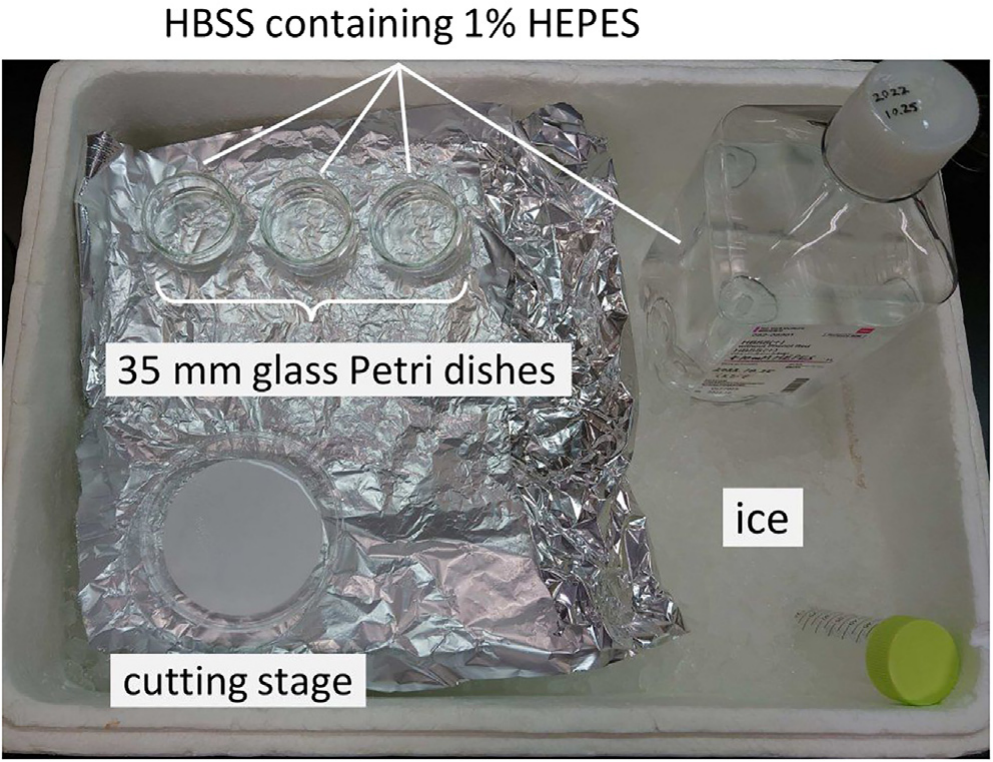
Suggested setup for a part of the working space 35 mm glass dishes are filled with HBSS containing 1% HEPES. Wash the dissected tissue a total of twice, once in each glass dish. Cut rods from the lung on a cutting stage with filter paper moistened with HBSS supplemented with 1% HEPES. Float the slices in the HBSS (+1% HEPES) in the third glass dish.
  c. Place the dishes with inserts on ice.
  d. Put a 10 mm plastic culture dish on ice upside down, place a circular filter paper (φ 7.0 cm) over it, and moisten it with HBSS (+1% HEPES). Use this as a cutting stage (Figure 6).
  e. Set up the chopping machine (McIlwain, Cat# 51350).
  f. Set the thickness to 300 μm and an appropriate sectioning speed.
  g. Attach a blade to the holder. Wipe the blade with 70% ethanol using a cotton swab.
  h. Layer two sheets of filter paper (φ 5.5 cm) on the circular stainless-steel table.
  i. Adjust the angle of the blade arm by test cutting the weighing paper until cut lines are visible in the center portion of the stage.

***Note:*** Detailed information about the chopper apparatus can be found at the link: http://www.limef.com/Downloads/McIlwain_manual.pdf

**Figure 7:**
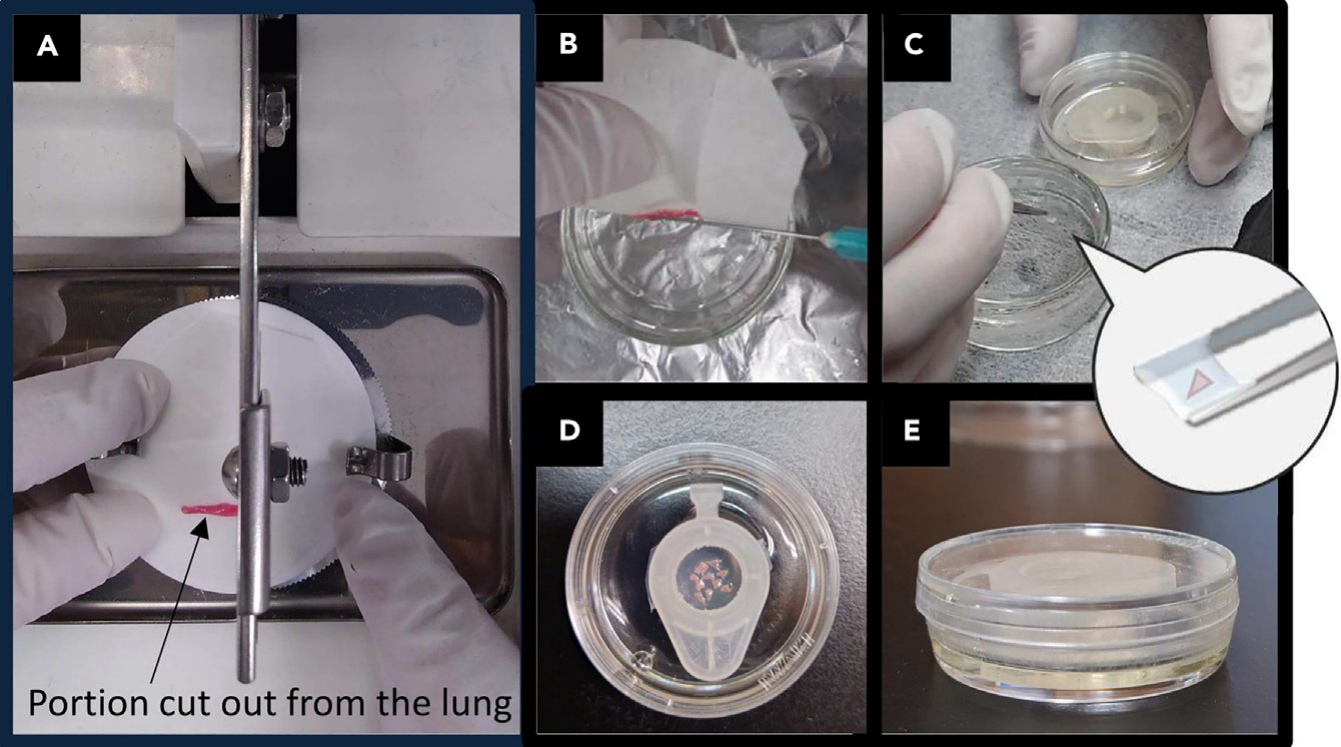
Procedure from cutting the slices to setting up the culture (A) Picture just before cutting the lung portion with the chopping machine. The rod of tissue cut out from the lung and the cutting arm are placed vertically. (B) The weighing paper is folded at the line the slices are on, and the slice is slid off the weighing paper using the side of the needle to float the slice in the HBSS (+1% HEPES) solution. (C) The slices are transferred to the handmade culture insert. The expanded image in the balloon shows how the slices are handled to avoid damage (see text). (D) Lung slices are placed on the handmade culture insert. (E) Dish containing lung slices sealed with Parafilm. Slices are cultured in this state at 37°C.

5. Prepare tools and solutions.
  a. Prepare the necessary tools for dissection (scissors, tweezers, razor blade and syringe with a 23G needle) and wipe them with 70% ethanol.
  b. Fill 35 mm glass Petri dishes with ice-cold HBSS containing 1% HEPES and place them on ice (Figure 6).

6. Administer anesthesia inhalation (isoflurane) to the mouse and euthanize it by cervical dislocation.
7. Spray the mouse with 70% ethanol. Incise the skin from the abdomen to the ribcage using scissors and cut the muscle to expose the abdominal cavity and organs.
8. Dissect the lung and wash it in the ice-cold HBSS containing 1% HEPES. Place it on the cutting stage.
9. Cut out a thin rod of tissue (1–2 mm width) from the edge of the lung with a razor blade and place it on the sterilized weighing paper.
10. Place the specimen on the weighing paper on the circular stainless-steel table of the chopping machine. Start cutting while fixing the paper with your fingers (Figure 7A).

**CAUTION**: This type of chopping machine does not have a guard around the cutting blade. Be very careful not to cut your fingers with the blade.

11. Using the side of the syringe needle, slide off the sliced lung from the weighing paper and float it in the ice-cold HBSS (+ 1% HEPES) solution (Figure 7B).
12. Place the slices one by one on the inserts so that they do not overlap. Finally, about 10 slices per insert should be placed (Figure 7D).

Pick up a slice very gently by lifting it so that it is held in the water column of buffer that forms between the tips of round-tipped tweezers without directly touching the slice (Figure 7C).

***Note:*** Since the quality of each slice will vary, the slices obtained from one rod should be placed randomly on each insert to average them out.

13. Observe each slice under a stereomicroscope. If it is folded, spread it out with the tip of a syringe needle so that it lies flat. Be careful not to damage the slice.
14. After all sample dishes are ready, seal the dishes with parafilm and place them in an incubator at 37°C to begin incubation (Figure 7E).

### Day 2: Circadian rhythm synchronization with DEX

**Timing: 2 h**

Just before initiating DEX stimuli, slices should be harvested for time point 0 samples according to the procedure described in the Day 2–4 step.

After approximately 24 h of incubation, synchronizing stimulation using DEX is conducted. This aligns the phases of the circadian rhythm of each cell of the slice, making the circadian rhythm stable and easy to observe in the time-course. Steps 16 and 18 should be conducted on a clean bench.

**CRITICAL:** A few hours before starting this step, place dishes containing medium for washing and re-culture in an incubator and keep them at 37°C before use. If the medium has been dispensed the previous day, bring it first to room temperature (20°C–30°C ) and then to 37°C.

15. Prepare 1.8 μL aliquots of 100 mM DEX in 1.5 mL test tubes for the number of samples.
16. Remove the samples from the incubator, and pipet a 500 μL portion of medium from the culture in the 1.5 mL test tube with DEX. Return the mixture of media and DEX to the dish and swirl it softly to mix (100 nM final concentration).

***Note:*** Collection of medium by pipetting is made easier by elevating the insert with tweezers.

17. Incubate the samples for 1 h at 37°C.
18. To wash out the DEX, put the inserts in dishes containing washing media. After all have been put in dishes, transfer them to dishes of re-culture medium, seal the dishes with parafilm, and restart incubation.

**CRITICAL:** After the inserts are transferred to the washing medium, swirling is not required. The washing process should involve moving the insert only and should not involve any unnecessary stimulus.

### Day 2–4: Harvesting tissue slices

**Timing: 2 days**

**Timing: 30 min at each time point (for steps 20–22)**

This section describes how to harvest and homogenize the tissue slices. Homogenization is the first step in RNA extraction.

**Figure 8:**
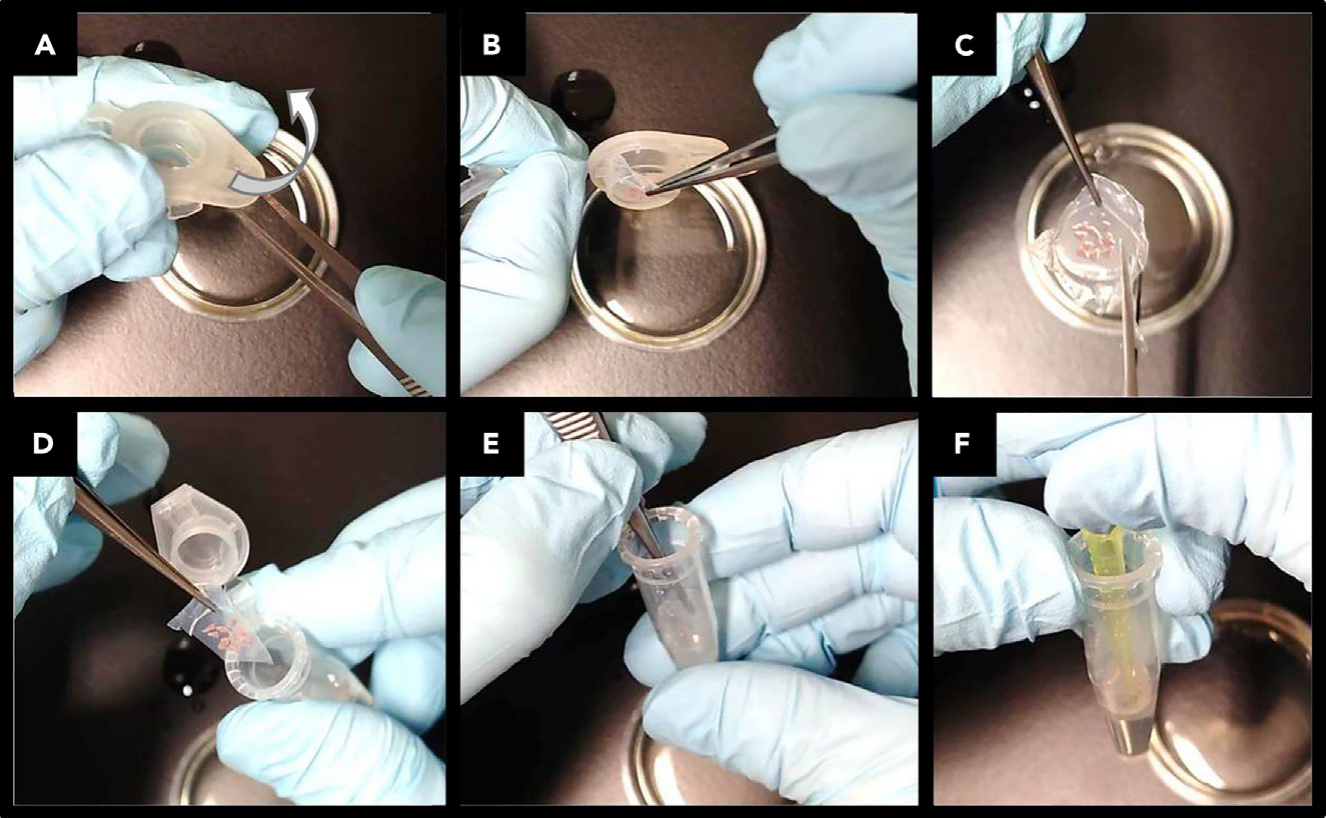
Harvesting the lung slices (A) Open the frame using round-tipped tweezers. (B) Peel off the membrane with the slice carefully. (C) Trim the membrane with scissors to fit the diameter of the test tube, avoiding the slice. (D and E) Soak the membrane in RLT buffer. (F) Scrape the slice from the membrane using a 200 μL pipette tip.
  b. Holding the membrane with the tweezers, trim the membrane with scissors to fit the diameter of the 1.5 mL tube containing RLT buffer (Figure 8C).
  c. Soak the membrane into the RLT (Figures 8D and 8E).
  d. Scrape off the slices using a 200 μL tip so that they float in the RLT (Figure 8F).

19. Dispense 300 μL RLT buffer (Qiagen) into the 1.5 mL test tubes with the textured inside of a BioMasher II (Nippi, Cat# 320102).
20. At harvesting time, harvest the lung slices.
  a. Open the insert frame and remove the membrane with the slice on it using tweezers (Figures 8A and 8B).

**CRITICAL:** Perform these steps quickly in move to prevent them from drying out.

***Alternatives:*** If using a commercial insert, cut out the portion of the membrane on which the slices are located and lyse them in the same manner as above.

21. Homogenize the slices well by pushing and turning the pestle supplied with BioMasher II approximately 30–50 times (Figure 9).

**Figure 9:**
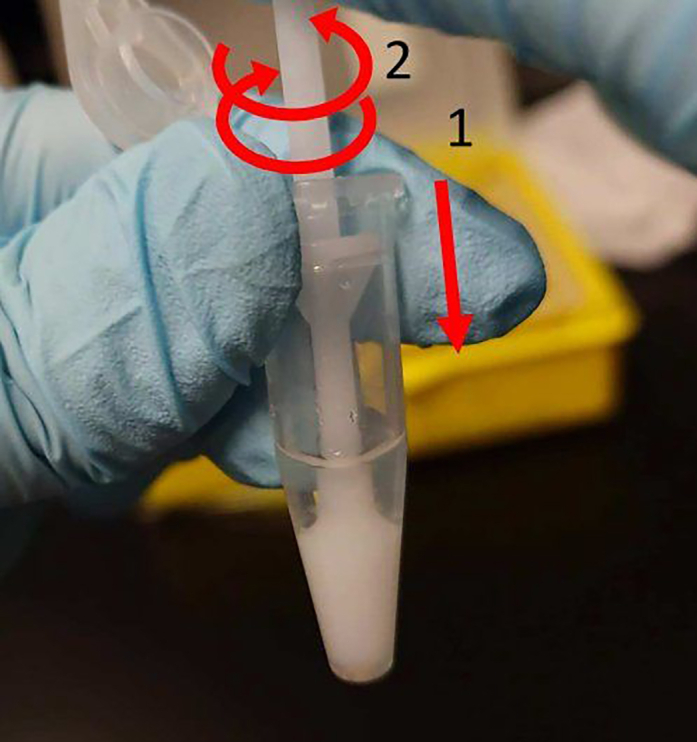
Homogenization of the harvested lung slices Push the pestle downward (1) and turn (2) to homogenize. Repeat steps 1 and 2 about 30–50 times.
22. Store the lysate samples at −80°C until the RNA extraction step.
23. Repeat this step every 6 h until all time-point samples have been collected.

**Pause point:** Lysates can be stored long-term at −80°C.

### RNA extraction and cDNA synthesis

**Timing: 1–2 days**

In the steps below, RNA is extracted from the lung lysate using an RNeasy Mini Kit (Qiagen) and complementary DNA (cDNA) is synthesized using a High Capacity cDNA Reverse Transcription Kit (Thermo Fisher Scientific).

24. To homogenize lung tissue completely, put the lysate prepared at the respective time points through a QIAshredder (Qiagen).
25. Extract RNA using the RNeasy Mini kit following the manufacturer’s instructions (RNeasy Mini Handbook - (EN) - QIAGEN).
26. Measure the concentration of extracted RNA in a spectrophotometer.
27. Perform reverse transcription to prepare complementary DNA (cDNA) using 1.5 μg of purified RNA obtained as described above. The reaction solution composition and conditions follow the manufacturer’s protocol (Document Connect (thermofisher.com)).

### Real-time quantitative PCR

This section describes the measurement of mRNA levels. The standard curve method is applied in this protocol, so a cDNA solution for standards and no-template control (NTC) are required. In addition, to confirm reaction specificity, this protocol also applies dissociation curve analysis after amplification (See Equipment setup for RT-q PCR).

28. Prepare the diluted cDNA samples and standards.
  a. Dilute the cDNA 5-fold by adding 80 μL dH_2_O since the total volume of the solution for the reverse transcription reaction described above is 20 μL.
  b. Dilute a known amount of vector cloning the cDNA of clock genes for standards. To ensure thorough and even coverage of your quantification range, sufficient dilutions should be prepared to cover the expected range of expression within your samples. In this protocol, we usually prepare a 5-point 10-fold serial standard with a range from 5 fg/μL to 50 pg/μL. For 18S-rRNA, the range is 50 fg/μL to 500 pg/μL.
29. Prepare the master mix for PCR reactions on ice by referring to the recipe in the materials and equipment section. See Table Sequences of primers used for real-time PCR.

**CRITICAL:** When calculating the master mix volume, ensure inclusion of the number of standards and NTC.

30. Dispense 20 μL of the master mix into each well of a 96-plate, add 5 μL of diluted cDNA, and mix well by pipetting on ice.

***Note:*** Use 96-well plates and lids that are appropriate for the real-time PCR equipment you use.

**CRITICAL:** Do not allow the formation of bubbles in the reaction solution on pipetting.

**CRITICAL:** After the cDNA addition of all samples is completed, check the wells for bubbles. Bubbles on the surface are no problem, but if bubbles occur in the liquid, move them to the surface by lightly tapping the bottom of the well.

31. When the plate is ready, seal it and set it in the real-time PCR detection system.
32. Run the RT-qPCR reaction. The equipment setup for RT-qPCR is described in the materials and equipment section.

## Expected outcomes

**Figure 10:**
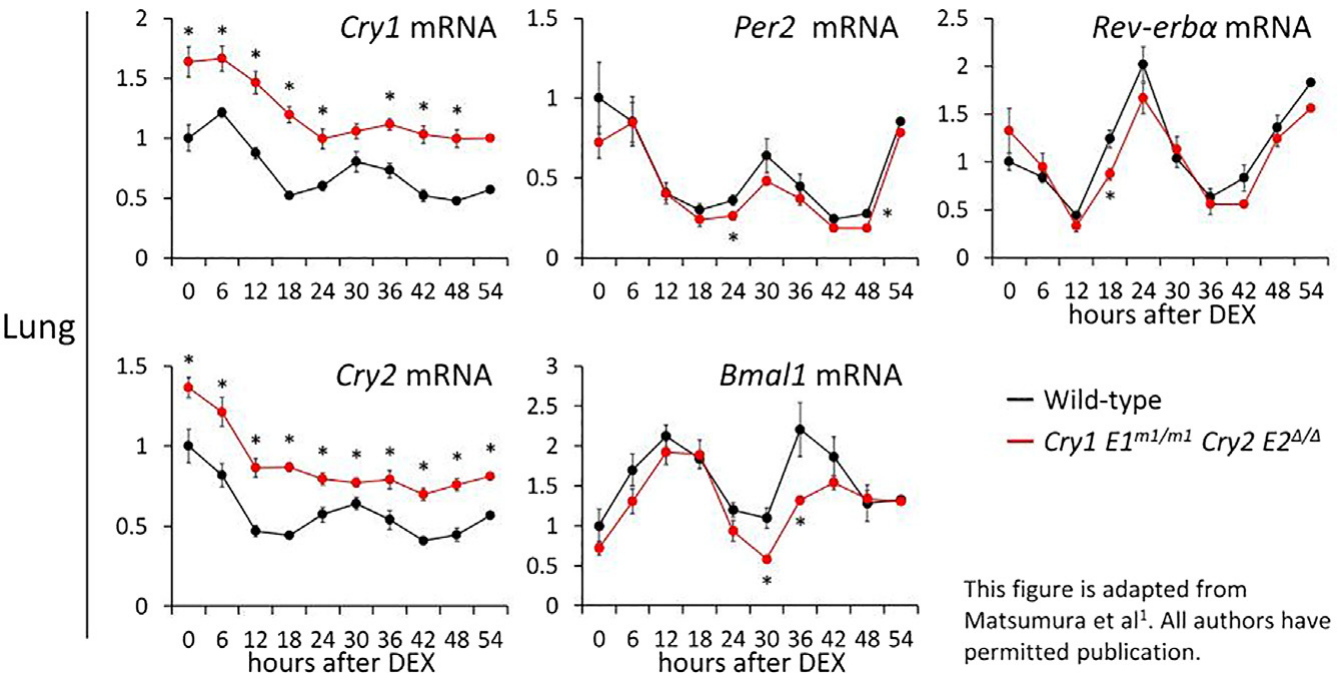
Endogenous circadian rhythms of clock gene mRNA expression in lung tissue slices from *Cry1 E1*^*m1/m1*^*Cry2 E2*^*Δ/Δ*^ double mutant mice detected by this protocol Plots indicate mean ± standard error of mean (n = 4 from 2 mice, ∗p < 0.05 by two-tailed t-test).

Successful completion of this protocol will allow observation of the rhythms of clock gene expression (Figure 10). In our study in which this protocol was applied,^1^ *Cry1, 2* mRNA data from *Cry1 E1*^*m/m*^ *Cry2 E2*^*Δ/Δ*^ double mutant mice, in which the loss of overt circadian transcription of *Cry* genes was induced by disrupting the E-box elements in their promoter regions necessary to produce circadian rhythm, served as both the target data and the arrhythmic control. If available, it is recommended to use mutant mice in which the rhythm is lost at the transcriptional level as a control (e.g., arrhythmic circadian transcription of the *Dbp* clock gene in *Bmal1*-KO mice). Alternatively, in the case that such mice are difficult to obtain, a good indicator in determining whether the detected rhythms are legitimate is by measuring the expression rhythms of clock genes that are in antiphase, such as *Per* genes and *Bmal1*.

It should be also mentioned that the amount of purified RNA obtained from lung slice samples per time point ranged from 33 to 196 ng/μL.

## Quantification and statistical analysis

RT-qPCR data should be normalized and quantified. Gene expression should be normalized to the reference gene 18S-rRNA. It helps to compare different genes if the first time point of the control (WT) is corrected as 1. Rhythmic or arrhythmic expression can be recognized visually, but it is better to identify a significant difference using the t-test at each time point (Figure 10). You can also use Cosinor provided by Refinetti to calculate the significance of rhythmicity.

## Limitations

Most molecular studies of circadian clocks measure circadian rhythms in clock gene mRNA or protein levels. This is because the substance of a circadian clock is the repetition of oscillation of clock genes in approximately one-day cycles of transcription and translation. When these changes over time are graphed, they form a cosine curve. The key components of a cosine curve are amplitude, phase, period length, and mesor. Excluding mesor, the remaining three elements are important measurements in circadian clock studies. This protocol can be used to determine whether a circadian rhythm is present or absent and the magnitude of its amplitude. However, an additional note is required concerning the phase of the detected rhythm. Namely, the circadian phase is reset by DEX and does not reflect the phase originally present in the mouse. Therefore, this protocol cannot be applied to experiments aimed, for example, at detecting differences in circadian phase in mice treated with phase-altering treatments. With proper mathematical analysis (i.e., cosine curve fitting) the period length can also be calculated. In fact, Cosinor, mentioned in the quantification and statistical analysis section, calculates the period length. However, the results should be accepted cautiously. Given that a 5-day-duration data set is considered reasonable for circadian rhythm experiments aimed at estimating period length,^5^ this protocol may not be suitable.

## Troubleshooting

### Problem 1

Trouble with the creation of handmade inserts (Step 3 in Before you begin).

### Potential solution

It may not be easy to create the stand portion of the insert. Our design serves as an example only, and other designs can be applied - the only provision is that the shape should ensure that the culture medium is spread under the membrane and that the medium components are sufficiently diffused. Ensure also that the frame is horizontal. Different kinds of tube can be used if they fit into a 35 mm dish. If the design is changed, the appropriate amount of medium should be determined; namely, the amount that allows the membrane to be fully wet with the medium without the inserts floating up. Of course, the use of commercial inserts is also a good option (before you begin).

### Problem 2

Enough slices cannot be made (Day1 step).

### Potential solution

For ease of handling, the portion of lung from which the slices are made is taken from the edge of the lung. However, if enough slices are not prepared, you can cut it out of a non-edge portion of the lung. We have used such slices previously and encountered no problems with them.

### Problem 3

Contamination in slice culture (Day1 step).

### Potential solution

The antibiotics (penicillin and streptomycin) supplemented in the culture medium are not effective against fungi, so if contamination occurs, colonies of fungi are observed on the slices in culture. To save workspace, we typically conducted the entire process from lung dissection to the preparation of slices in an area separated from the regular laboratory by drapes; however, this area is unsterile. Therefore, time for which the lid of the culture dish containing the culture insert is opened should be minimized to lower the risk of contamination. Remember to clean the tools used for slice preparation with 70% ethanol. Also, workers should remember to wear gloves and masks. If contamination occurs, consider performing the work inside a clean bench.

### Problem 4

Unexpected trouble during slice harvesting (Day 2–4 step).

### Potential solution

Slice harvesting operations do not always go smoothly. Although we recommend practice beforehand, unexpected troubles may occur. For example, you may not be able to trim the membrane well or the edge of the membrane may stick to the slice; even in such cases, soak the membrane with the slice on it in RLT buffer anyway before the slice dries. This will avoid RNA extraction failure.

### Problem 5

Amount of extracted RNA is low (RNA extraction step).

### Potential solution

One troubleshooting procedure is to increase the number of slices placed on an insert. Alternatively, it is possible that the slices are damaged, possibly due to the poor condition of the sectioned slices or poor culture conditions. Slices should be prepared as quickly as possible and handled very gently. Also, the culture dish should be kept on ice until the start of culture and the medium for DEX washing and re-culture should be warmed before use (Day 1 and 2 section). As a preliminary experiment, we recommend seeing if the amount of RNA is within the indicated range in the expected outcome section following extraction from cultured slices for 24 or 48 h (expected outcomes). In our experience, RNA yields tend to increase with incubation time.

## Resource availability

### Lead contact

Further information and requests for resources and reagents should be directed to and will be fulfilled by the lead contact, Makoto Akashi (akashima@yamaguchi-u.ac.jp).

### Materials availability

This study did not generate new unique reagents.

## Data Availability

All data reported in this paper will be shared by the lead contact upon request. Any additional information required to reanalyze the data reported in this paper is available from the lead contact upon request.

## Supplemental information

Table S1. Sequences of primers used for real-time PCR, related to step 29
